# Highlight: Not Like a Textbook—Nuclear Genes in *Blastocystis* Use mRNA Polyadenylation for Stop Codons

**DOI:** 10.1093/gbe/evu167

**Published:** 2014-08-07

**Authors:** Danielle Venton

When we peer into the overlooked, underexplored corners of biology, mysterious, unexpected forms of diversity tend to reveal themselves. Our understanding of the workings of life unfolds a little more as a result.

A team of researchers from Canada and the Czech Republic recently found that the eukaryote *Blastocystis*, a common human parasite, shares genetic traits previously known only in mitochondria. Their results are published in *Genome Biology and Evolution* (Klimeš et al. 2014).

“Whenever people find things that do not fit into our [standard] philosophy, it opens our minds,” says Gertraud Burger, a biologist studying the evolution of the prokaryotic genome at the University of Montreal who was not involved in the work.

More than 30 years ago Stephen Anderson at Cambridge University and coworkers first described an unusual way for messenger RNA in mammalian mitochondria to signal it was time for translation to stop. Part of the termination codon UAA (UAA is one of several stop codons) was created by polyadenylation—the addition of multiple adenosine monophosphates bases to RNA. The same trait has since been spotted in the mitochondria of other organisms, such as dinoflagellates and other flagellate protozoa. For this trait to be found in nuclear genomes, however, the work of Klimes et al. (2014) marks a first.

“We tend to see strange things in pathogens and endosymbionts—they have a fast evolution and invent curious things. Still,” says Burger, “finding incomplete stop codons is not expected.”

“It is very surprising,” says coauthor Marek Eliáš, of the University of Ostrava in the Czech Republic. “Normally the textbook view of the mechanism of gene expression is, the coding sequence which is interpreted by the ribosome is fully specified by the gene sequence.”

That assumption, he says, underlines programs such as Augustus or EuGene that are used to predict genes in newly sequenced genomes.

“Normally when you want to deliminate genes you have a normal nucleotide sequence, the program looks at the sequence and tries to figure out which are coding sequences and which are noncoding sequences, what are introns what are exons and so on,” says Eliáš. “The program assumes that there must be a termination codon that completes the coding sequence.”

In the case of *Blastocystis*, however, much of the annotated sequence submitted to GenBank, an open access sequence database, by prior teams is likely incorrect. In this work, Eliáš and coworkers defined the polyadenylation sites for nearly 2,500 nuclear genes. In about 15% of these genes, termination codons were created by adding a poly(A) tail at a spot where the underlying gene sequence did not stipulate a stop codon.

Might these have merely been a mistake? “In virtually all these genes,” write the authors, “there was no evidence for an alternative form of the 3′-end of the transcript, suggesting that the introduction of the termination codons by polydenylation does not result from processing of aberrant or incomplete transcripts.” It does mean, however, that the molecular machinery at work is exceptionally precise.

“Apparently *Blastocystis* has a very accurate system for specifying the exact position, the exact nucleotide where the original transcript is cleaved and the adding of those adenosines start,” says Eliáš.

Ross Waller, a biochemist at the University of Cambridge not involved in the study, says the work of Eliáš and coworkers is an excellent reminder that to fully understand the molecular biology of cells, it is important to study diverse eukaryotes. “It also points to the limitations,” he says, “of bioinformatics where inherent assumptions of an analysis are not questioned—in this case that genomic coding sequences will be defined by a translation termination codon.”

The finding came unexpectedly. Members of the team are interested in studying GTPases. “I know this group of genes so well,” says Eliáš, “I could immediately see that there is something strange in *Blastocystis*.” He noticed that the genes were lacking an expected motif typical of the family. “That is why we were able to find something completely novel.”

Once they factored in the termination codons created in part by poly(A) tails, the predicted proteins do bear the prenylation motifs Eliáš was expecting. There is a lesson here, says Burger, for researchers in paying attention to detail.

“Perhaps we [all] have to be more critical of what we find,” she says. “If it does not fit our hypothesis we should look for the reason, instead of putting it aside and thinking ‘It’s an error’ or ‘The analysis wasn’t well done’.”

Now, Eliáš and his coworkers are reannotating one of the *Blastocystis* genome sequences currently in GenBank. Once completed, this will allow for better gene models. Eliáš believes that it is unlikely the discovery will create ripples in the medical world. The machinery responsible for synthesizing a polyA tail and completing the termination codons, he postulates, is likely general cellular machinery. So using it to find drugs that are specific for *Blastocystis* without having negative effects on the human hosts is unlikely.

“For me the most critical thing was to notice the phenomenon,” he says. “I think the community will view this as another example of an unorthodox behavior from an obscure eukaryotic lineage.”

The farther and deeper we look, he says, the less life looks like a textbook.
